# Improved Mechanical Properties of Polyurethane-Driven 4D Printing of Aluminum Oxide Ceramics

**DOI:** 10.3390/ma18081750

**Published:** 2025-04-11

**Authors:** Zhaozhi Wang, Zhiheng Xin, Zhibin Jiao, Chenliang Wu, Xu Bai

**Affiliations:** 1 School of Mechanical Engineering, Shenyang University of Technology, Shenyang 110870, China; zhaozhi_wang@sut.edu.cn (Z.W.); xinzhiheng662@163.com (Z.X.); baix@sut.edu.cn (X.B.); 2 School of Materials Science and Engineering, Shenyang University of Technology, Shenyang 110870, China; mfclwu@sut.edu.cn

**Keywords:** 4D printing, self-deforming ceramics, PDMS, polyurethane, thermal decomposition, aluminum oxide, mechanical property optimization

## Abstract

The current deformation scheme used in the 4D printing of ceramics has several disadvantages, such as a poor deformation capacity, high process complexity, and the poor mechanical properties of the product. In order to solve these problems, the deformation scheme introduced in this study utilizes the pyrolytic expansion of polyurethane and the resulting pores to hinder the contraction of the specimen during the ceramization stage. Then, the specimen is composited with a polyurethane-free portion that has a high rate of shrinkage, and deformation is initiated through the interlayer stress mismatch generated by the difference in the shrinkage of the different layers, thus enabling the preparation of complex structural ceramics. This solution is simple and efficient; heat treatment is performed in a single pass, and the precursor specimen is highly deformable. The incorporation capacity of the aluminum oxide ceramic powder was increased by replacing part of the Dow Corning SE 1700 polydimethylsiloxane silicone rubber in the raw material with Dow Corning DC 184 polydimethylsiloxane silicone rubber, which, in turn, improved the mechanical properties of the obtained ceramics by enhancing the solid-phase content of the ceramic powder. Due to the introduction of polyurethane, the ceramic has a secondary pore structure, which has the potential for application in the field of engineering materials and heat insulation materials.

## 1. Introduction

Four-dimensional printing is an advanced additive manufacturing technology that combines 3D printing and stimulus-responsive smart materials. The fourth dimension is described here as transformation over time [1]. This technique builds performance-driven functionality into the materials. Specifically, the object’s shape and performance can undergo further modification over time by response to external stimuli, such as optical [2], thermal [3], and various environmental conditions [4,5,6]. This process enables functions such as automatic deformation, self-repair, and autonomous assembly [7]. In order to realize the 4D deformation of the material, it is necessary to introduce suitable actuating materials into the material in response to stimuli from the external environment. The materials applied in 4D printing cover many fields [8], such as photosensitive resins [9], shape-memory polymers [10,11], hydrogels [12,13], metals [14], and ceramics. Materials with biocompatibility [15], biodegradability [16], reversible stretchability [17], high sensitivity [18], tunable electrical properties [19], and suitable mechanical properties [20] can be used in 4D printing, with potential applications in a variety of fields, such as the biomedical field [21,22,23,24,25], the food industry [26,27], the electronics industry [28,29], soft robotics [30,31], and aerospace [32]. Ceramic materials are widely used in aerospace, precision machinery, biomedicine, electronic technology, and many other fields [33,34]. However, due to their high melting point, hardness, and brittleness, it is difficult to realize certain complex forms of ceramics using traditional processing techniques. The concept of 4D printing provides a new possibility for the preparation of complex forms of ceramics [35]. Existing methods for the 4D printing of ceramics mainly consist of printing elastic materials into ceramic precursors with elasticity using direct ink writing (DIW) or stereo lithography apparatus (SLA) and then shaping the elastic precursors into the desired morphology via physical means, such as origami, pre-stretching, and so on [36]. In this way, the deformation of ceramic precursors and ceramization are carried out separately, which reduces the fabrication efficiency. Furthermore, during the sintering process of the ceramic precursor from polymer to ceramic state, the introduction of fixtures may result in a local shrinkage mismatch, which may consequently trigger an uneven stress distribution or even crack generation. Alternatively, the composite printing of slurries with different material ratios can be utilized to achieve the bending deformation of ceramic materials through the different shrinkage rates of the precursors after sintering [37], and the bending direction of the ceramics can also be controlled by varying the print paths during the printing process [37]. The 4D deformation of ceramics can also be realized by leveraging the memory ability of shape-memory polymers [38]. Alternatively, multiple materials can be composited to drive precursor deformation with materials that can deform violently in characteristic environments, such as using hydrogels to drive precursor deformation [39]. These deformation-inducing polymeric materials will be decomposed during the subsequent sintering, thereby losing the function of fixing the structure and triggering a re-verse deformation. In the process of printing precursors, parameters such as print speed, extrusion pressure, print height, layer thickness, nozzle diameter, and so on can influence the deformability of the precursors [18,40]. To date, porous materials such as expanded polystyrene (EPS), polyurethane (PU), polyethylene (PE), polypropylene (PP), and other porous materials have been used to prepare porous ceramics [41], among which PU has better flexibility than the other materials with a controllable pore structure [42]. Thus, it can be used as a precursor for the preparation of elastomeric precursors. The lower thermal decomposition temperature of polyurethane ensures that it can complete the pore-making process before the thermal decomposition of polydimethylsiloxane (PDMS) occurs, thus realizing the deformation behavior of the specimen. Therefore, polyurethane was introduced as the pore-making material to drive the deformation of ceramic precursors in this study.

To realize the self-deformation of complex structural ceramics, this study presents an innovative ceramic 4D printing approach based on a polydimethylsiloxane–polyurethane (PDMS-PU) composite ink system, which is simple, efficient, and can significantly improve the deformation ability of ceramic precursors. A 4D printing slurry was prepared by mixing PDMS, alumina nanoparticles (Al_2_O_3_), and different amounts of polyurethane. Double-layer composite structures were then printed using a direct-write printer, with each layer printed using slurries with different amounts of PU added. PU undergoes thermal decomposition during the sintering process to create pores inside the precursor, which, in turn, allows the specimen to expand. During subsequent ceramization, the pores impede the shrinkage of the precursor, further reducing the shrinkage rate, which creates an internal stress mismatch when it is composited with a high-shrinkage material, driving the bending deformation of the material. The volume change rule of ceramic precursors with different PU contents in different heat treatment stages was investigated, and the effect of the PU content on the ability to induce self-deformation of lamellar ceramic precursors was tested and analyzed. This method improves the ceramic deformation capability without requiring additional auxiliary tools and external environmental stimuli, retaining the simple and efficient advantages of active deformation. Its self-deformation and ceramization sintering can be completed in one heat treatment process, which improves the preparation efficiency. Through this deformation method, several complex ceramic structures with different shapes, such as scorpion (localized deformation, controlling the deformation of some positions of the specimen) and octopus (a bi-directional curled structure; one part of the specimen realizes bending in two opposite directions) shapes, were designed and fabricated to verify the feasibility of the scheme.

PDMS DC 184 is a silicone elastomer widely used in microfluidics, flexible electronics, and biomedical applications. In comparison to SE 1700, DC 184 exhibits superior flowability, enabling the incorporation of a greater quantity of ceramic powder and allowing for an increase in the mass fraction of the ceramic powder using the same printing parameters, which, in turn, improves the mechanical properties. The feasibility of the scheme was verified by replacing part of the SE 1700 with DC 184 to increase the fluidity of the slurry, thus increasing the solid-phase content of the ceramic powder and improving the mechanical properties of the ceramics obtained after sintering. The feasibility of the scheme was also verified by comparing the produced material with the original slurry specimen using the three-point bending test. The optimization scheme retains the advantages of using polyurethane pyrolysis to drive the 4D deformation of ceramics, and the test specimen can be directly deformed to the final form in one heat treatment. The fluidity of the slurry can be improved by optimizing the ratio of materials in the slurry, and then by increasing the incorporation capacity of the ceramic powder to improve the mechanical properties of the ceramic specimens, which is of great significance for the application of 4D-printed ceramics in engineering.

## 2. Materials and Methods

### 2.1. Materials

Two polydimethylsiloxanes (PDMS SE 1700 and PDMS DC 184), purchased from US Dow Chemical Company (Midland, MI, USA), were blended with a silicone base (Vinyl capped PDMS) and a curing agent (compounds containing silicon–hydrogen bonds) in a 10:1 mass fraction; polyurethane (PU) was purchased from China Shenzhen Ausbond Co., Ltd. (Shenzhen, China), and blended with a curing agent (polyols and diamines) with a mass fraction of 5:1. Lipophilic Al_2_O_3_ nanoparticles with a diameter of about 50 nm were purchased from China Zhongke Xincai (Beijing, China) and dried in a vacuum drying oven for 6 h at 100 °C before use.

### 2.2. Preparation of Precursor Slurry

The preparation of the base slurry (Type I slurry) was as follows: The PDMS (SE 1700) silicone base material and curing agent were added to a container at a ratio of 10:1 by weight and mixed with a glass rod for 5 min. The mixed PDMS and Al_2_O_3_ nanoparticles were added to the container at a ratio of 2:1 by weight and mixed with a glass rod for 20 min, which was used as the Type I base slurry. The polyurethane base material and curing agent were added to the container at a weight ratio of 5:1 and mixed and stirred with a glass rod for 5 min. Given that PU was introduced into the slurry as a foaming material and pyrolysis volatilization occurred almost completely during the sintering process, and given that the density of PU is much higher than that of PDMS, the decision was made to incorporate PU into the slurry as an additional introduction in order to ensure that the ceramic composition obtained by sintering remained unchanged. The mixed PU was added to the mixed base slurry; the mass of the added PU was 1:6, 1:3, or 1:2 of the mass of the mixed PDMS and Al_2_O_3_ nanoparticle mixture (base slurry). The slurry was stirred with a glass rod for 20 min and then transferred into a barrel. This slurry was used as the Type I deformation slurry.

The preparation of control group slurry (Type II slurry) was as follows: SE 1700 and DC184 were mixed individually. After mixing the respective body and curing agent in the ratio of 10:1 by mass, the two mixed base materials were combined at a mass ratio of 2:1 and stirred. The mixed base materials and Al_2_O_3_ nanoparticles were added to a container at a mass ratio of 2:1 and stirred with a glass rod for 20 min, and then the slurry was used as the Type II base slurry. The mixed polyurethane was added to the mixed base slurry; the mass of the added polyurethane was 1:6, 1:3, or 1:2 of the mass of the Type II base slurry. The slurry was stirred with a glass rod for 20 min and then transferred into a barrel. This slurry was used as the Type II deformation slurry.

The preparation of modified slurry (Type III slurry) was as follows: To improve the mass fraction of Al_2_O_3_ nanoparticles in the Type II slurry, a well-mixed slurry of SE 1700 and DC184 and Al_2_O_3_ nanoparticles were added to a container at a mass ratio of 1:1. The slurry was mixed and stirred with a glass rod for 20 min and then used as the base slurry for the Type III slurry. The well-mixed polyurethane was added to the well-mixed base slurry; the mass of the added polyurethane was 1:6, 1:3, or 1:2 of the mass of the Type III base slurry. The slurry was mixed with a glass rod for 20 min and then transferred into a cylinder. This slurry was used as the Type III deformation slurry.

Before printing, the cylinder filled with slurry was centrifuged in a planetary centrifuge at 1000 rpm for 10 min for defoaming. It could then be placed in the printer for printing.

### 2.3. Rheology Analysis of Slurry

The rheological properties of the precursor slurries were measured on a rotational rheometer (Kinexus Prime pro+ produced by China NETZSCH Scientific Instruments Trading (Shanghai) Ltd., Shanghai, China). Viscosity was measured at 32 °C in flow mode with shear rates ranging from 10^−4^ to 300 s^−1^ and shear strains ranging from 0.2 to 10,000%. In order to measure the differences in the rheological properties of the three slurries, the three sets of base slurries were configured for testing.

### 2.4. Three-Dimensional Printing

The precursor slurry was printed using the DIW (Bio-Architect^®^SR produced by China Hangzhou Regenovo Biotechnology Co., Ltd., Hangzhou, China) printing method. Due to the high viscosity of the slurry, a screw extrusion printing module was used for printing, with a print nozzle diameter of 0.51 mm, a print layer height of 0.4 mm, a print filament spacing of 0.8 mm, a screw extrusion speed of 0.085 mm/s, and a print speed of 3.5 mm/s. Each layer of the print path was deflected by 90° compared with the previous layer. The printed precursor structure was cured in a drying oven at 80 °C for 2 h to form a complete crosslinking network of the precursor, thus completing the preparation of the elastic precursor.

### 2.5. Sintering

The cured ceramic precursors were sintered in a muffle furnace (FZQ-12/16 produced by China Facerom Thermal Equipment Co., Ltd., Hefei, China) with an atmosphere of 99.99% pure argon. To ensure the complete thermal decomposition of the added polyurethane, PDMS does not undergo thermal decomposition at this time, the sintering temperature should be maintained for 2 h when sintering to 400 °C. The sintering process was as follows: the temperature was increased from room temperature to 400 °C at a rate of 1 °C/min and held for 2 h to ensure the initial pyrolysis of PU [43]; it was then increased to 800 °C at 1 °C/min and held for 2 h; then, it was increased to 1350 °C at 1 °C/ min and held for 2 h to obtain a tight ceramic structure [4]; finally, it was decreased to room temperature at a rate of 1 °C/ min, yielding the dense alumina ceramics.

### 2.6. Characterization

In order to observe the microstructures of the ceramics, 10 mm × 10 mm × 5 mm cuboids were printed, cut into sections, and sintered. The microstructures of the printed structures were visualized using scanning electron microscopy (SEM, GEMINISEM 300 produced by German Carl Zeiss AG, Oberkochen, Germany) at magnifications of 40× to 5000×. A lower magnification was used to observe the macroscopic structure of the ceramic surface, which was used to determine the macroscopic effect of the introduction of PU on the ceramic structure. A high magnification was used to observe the microstructure of the ceramic surface and interior to determine the effect of PU introduction on the ceramic porosity. The elemental composition of the ceramics was determined by facet-scanning selected areas with an energy dispersive spectrometer (EDS, produced by German Carl Zeiss AG). The peak distribution of the sintered ceramics was examined using X-ray diffraction (XRD, Bruker D8 advance, produced by German Bruker Corporation, Bremen, Germany) and compared with the peaks of standard specimens in the database to determine the main composition of the ceramics. The test specimens were crushed with a mortar and sieved through a 200-mesh sieve for the experiment. The XRD experimental parameters were as follows: a scanning angle of 5–90° and a scanning speed of 2°/min.

Fourier infrared absorption spectroscopy (FTIR, Thermo Nicolet IS5, produced by US Thermo Fisher Scientific, Waltham, MA, USA) was used to determine the functional group species during the curing process. The measurement of the functional group species after curing required the preparation of a block specimen with a relatively flat surface and a size of 20 mm × 10 mm × 2 mm using a print nozzle diameter of 0.51 mm, a print layer height of 0.4 mm, a print filament spacing of 0 mm, a deflection angle of 90 ° for each layer, attenuated total reflection mode, and a test wave number of 600–4000 cm^−1^.

### 2.7. Mechanical Performance Test

A universal testing machine (UTM6000 produced by China Shenzhen Suns Test Technology Co., Ltd., Shenzhen, China) was used to perform a three-point bending test on the ceramic specimens, with a support span of 24 mm and a bending displacement rate of 0.5 mm/min. Rectangular specimens with a size of 50 mm × 5 mm × 5 mm were printed to measure the mechanical properties of the ceramic specimens. Three tests were performed for each type of specimen.

## 3. Results

### 3.1. Basic Deformation Principles

**Figure 1:**
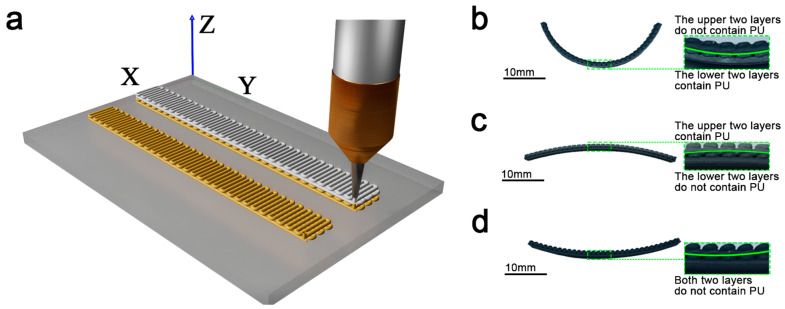
(**a**) The printing of precursors. (**b**) The paste containing PU was printed on the lower two layers, and the base paste without PU was printed on the upper two layers; the bending morphology of ceramic specimens was obtained by sintering in order to clearly show the printed structure, the green line box shows a partial enlarged view of the specimen, the same as (**c**,**d**). (**c**) The paste not containing PU was printed on the lower two layers, and the base paste containing PU was printed on the upper two layers; the bending morphology of ceramic specimens was obtained by sintering. (**d**) Printed with PU-free paste only; the bending morphology of ceramic specimens was obtained by sintering.

Figure 1 depicts the print preparation process of the programmable precursors and presents a schematic diagram of the self-deformation that occurs during sintering. In order to investigate the deformation law of the precursor, the specimen was printed with four layers, and the lower two and upper two layers were printed with different slurries, as shown in Figure 1a. Figure 1b and Figure 1c compare the bending directions and curvatures of the precursors after sintering with the same two slurries printed, but with a different lamination order. The same precursor structure was printed with only the PU-free Type I slurry, and the curved structure in Figure 1d was obtained after sintering into ceramics. With this printing path, a long strip of precursor will tend to warp upward and bend in the process of sintering. The PU content affects the porosity of the ceramic specimen, and a change in porosity changes the specimen’s strength and thermal conductivity. Therefore, two different printing methods can be used to make ceramics with the same curvature but with different porosities, resulting in a wide range of options for different physical properties in practical applications.

**Figure 2:**
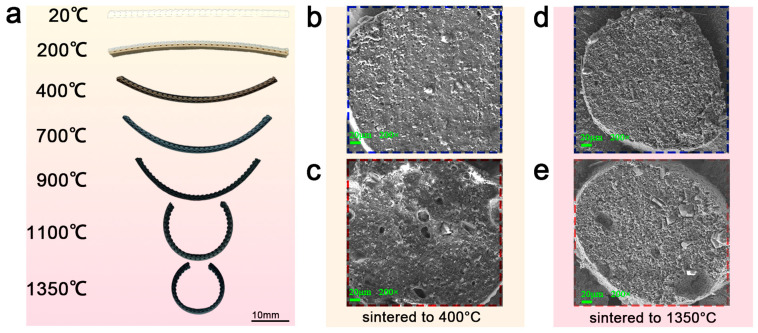
Morphological changes in precursors during sintering. (**a**) The deformation morphology of precursors at various temperatures. (**b**) Microstructure of PU-free specimens when sintered to 400 °C. (**c**) Microstructure of PU-containing specimens when sintered to 400 °C. (**d**) Microstructure of PU-free specimens when sintered to 1350 °C. (**e**) Microstructure of PU-containing specimens when sintered to 1350 °C.

A basic deformation specimen with a size of 50 mm × 5 mm × 1.6 mm was printed and divided into two parts, with two layers in each part, and the print path between the layers was deflected by 90°. The upper part was printed with the Type I basic slurry, and the lower part was printed with the Type I slurry with the 1/3 PU addition. The changes in specimen shape were observed at different temperatures in a protective Ar gas atmosphere to characterize the deformation process of the specimens, as shown in Figure 2a. It can be observed that the PU in the specimen fully decomposed when the sintering temperature was increased from room temperature to 400 °C, generating a large amount of gas and forming a porous structure within the material (Figure 2b,c). The gas generated by the pyrolysis of PU causes the PU-containing portion to expand, which induces the bending of the specimen toward the opposite side. As the temperature continued to increase, the overall shrinkage rate decreased, but as the specimen side containing PU contained a large number of pores, these pores impeded the shrinkage of the specimen in that side, which resulted in a lower shrinkage rate. This generated an interlayer stress mismatch, which increased the bending curvature of the deformation of the specimen until the sintering temperature reached 1350 °C, resulting in the formation of a dense ceramic (Figure 2d,e). Utilizing this deformation principle, a composite of precursors with different PU contents causes a stress mismatch in the sintering process, which drives the bending deformation of the specimen.

**Figure 3:**
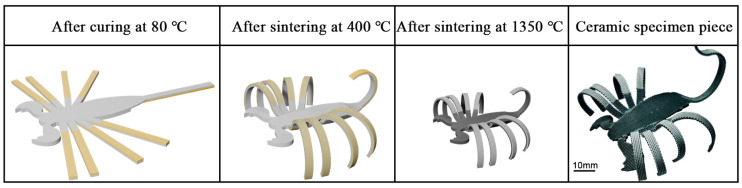
The realization of complex printed structures.

Because the curvature can be statistically varied by adding different amounts of PU, the ceramic structure is programmable. Thus, the 4D printing of complex ceramic structures can be realized, as shown in Figure 3.

### 3.2. Basic Deformation Laws and 4D Printing of Deformed Ceramics with Complex Shapes

**Figure 4:**
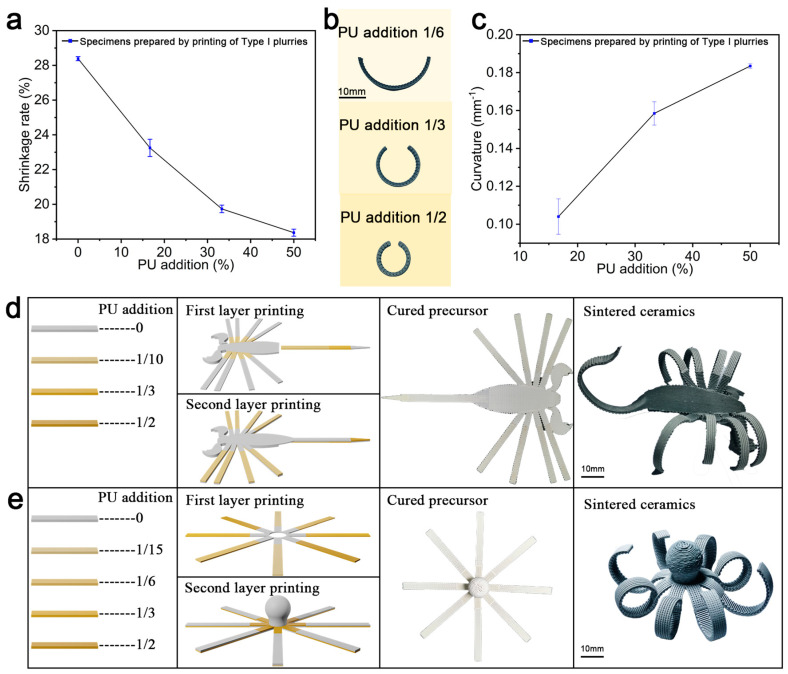
The basic deformation law and preparation of ceramics with complex morphologies. (**a**) The variation rule of the extra amount of PU added and the shrinkage rate of ceramic specimens prepared with the Type I slurry. (**b**) The bending morphologies of ceramic specimens prepared with the Type I slurry with different PU contents and printed on the lower two layers. (**c**) The law of change in the bending degree of ceramic specimens prepared with the Type I slurry. (**d**) The preparation of ceramics with a scorpion morphology. (**e**) The preparation of ceramics with an octopus morphology.

In order to design and prepare self-deforming ceramics with complex structures, it is necessary to investigate the self-deformation law of ceramic specimens. A rectangular specimen of 50 mm × 5 mm × 5 mm was printed with the Type I slurry to measure the shrinkage rate of the sintered precursor printed with each slurry. Figure 4a shows a graph of the calculated shrinkage rate variation, which illustrates that the shrinkage rate of the sintered precursor decreased significantly with the increase in polyurethane content. In order to print the basic deformation specimen, the lower two layers were printed with the Type I deformation slurry, the second layer was deflected by 90° with respect to the printing direction of the first layer, the upper two layers were printed with the Type I base slurry, and the fourth layer was deflected by 90° with respect to the printing direction of the third layer. A comparison of the curvature of the ceramics after sintering using this layer stacking method is shown in Figure 4b. Figure 4c shows the measurements of the bending morphologies in Figure 4b, showing that the curvature gradually increased with the increase in polyurethane content, which is in line with the change rule of the related slurry stretching rate in Figure 4a. Thus, self-deforming ceramics with complex structures can be designed to obtain different target geometries by adjusting the content of incorporated PU, as demonstrated in the plot of PU incorporation versus the curvature of the specimen in Figure 4c. Scorpions and octopuses with different curvature variations were fabricated to test the ability of this 4D printing scheme to prepare complex shapes. The legs of the scorpion in Figure 4d were printed using the addition of PU at a 1/10 mass for the substructure; the substructures of the scorpion’s tail, body, and pincers were printed using the Type I base slurry, and then the superstructure of the scorpion’s tail was printed using slurries with different PU contents to drive the gradient curl of the scorpion’s tail. The octopus tentacles in Figure 4e were divided into two segments, which were printed with different composite sequences to realize the bi-directional bending of the octopus tentacles. The preparation of these two self-deforming ceramics with complex morphologies verifies the programmability and controllability of the self-deforming scheme.

### 3.3. Optimization of Curing Reaction Process of Slurry and Ceramic Precursor for 3D Printing

**Figure 5:**
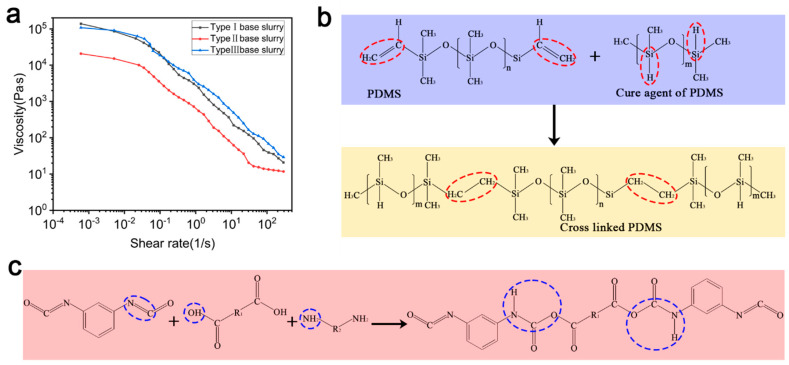
The characterization of 3D-printed slurries and the printing precursor properties. (**a**) Viscosity versus shear rate for three types of base slurries. (**b**) The PDMS curing process, the red circles show the relevant functional groups involved in the reaction. (**c**) The PU synthesis and curing process, the blue circles show the relevant functional groups involved in the reaction.

**Figure A1:**
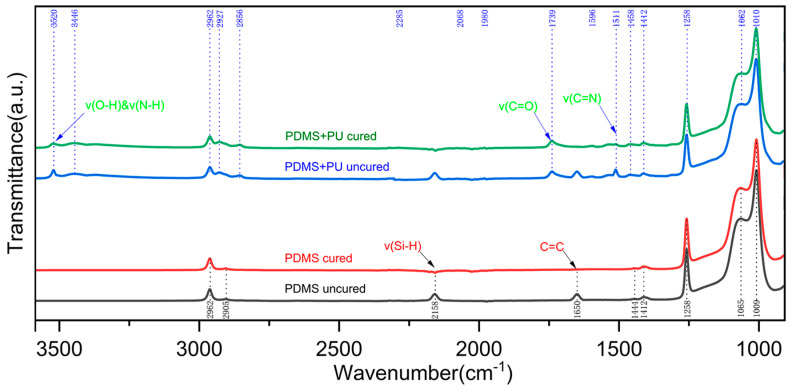
FTIR curves of PDMS and slurry before and after curing with PU addition.

The blending of a suitable precursor slurry for 3D printing is key to realizing precursor printing. Figure 5a shows the curves of viscosity versus the shear rate for the three types of base slurries. SE 1700 was solid before curing, and its incorporation capacity in the ceramic powder was limited. When the solid-phase content of Al_2_O_3_ reached 33%, it had basically reached its dissolution limit, and the viscosity of the slurry was very high at this time. Conversely, DC184 was liquid and had a higher incorporation capacity in the ceramic powder, but too much DC184 would increase the adhesion of the slurry during thinning, which would negatively affect the printing and preparation of the precursor, so it was necessary to control the amount of DC184 introduced. The ceramic slurry of pure SE 1700 (black line) exhibited shear-thinning behavior, and with the addition of DC 184 (red line), the viscosity of the slurry decreased, but its shear-thinning behavior did not change, which increased the fluidity of the slurry and the incorporation capacity of Al_2_O_3_ nanoparticles in this state. After increasing the solid-phase content of Al_2_O_3_ to 50 wt% (blue line), the viscosity of the ceramic slurry was greatly improved. At low shear rates, ceramic slurries of pure PDMS had viscosities as high as 1.38 × 10^5^ Pa·s, and slurries at this viscosity can be printed by using screw extrusion printing methods. During the curing process of PDMS, the C=C bond reacted with the Si-H bond of the curing agent to form a crosslinked network. The chemical formulas of the reactants and product are shown in Figure 5b, which is verified by the significant decrease in the absorption peaks located at 1650 cm^−1^ versus 2158 cm^−1^ in the curves for PDMS before and after curing in Figure A1. Figure 5c shows the curing reaction process for polyether-type polyurethane, whose raw materials are mainly isocyanate and polyether polyol, with diamine as a curing agent: isocyanate was used as a hard segment to provide rigid curing, polyether polyol was used as a soft segment to provide flexibility, and diamine was used as a chain extender and a curing agent. The C=N bond in diisocyanate reacted with the H-O group in the diol and the H-N bond in the diamine, respectively, to form a long-chain structure in the process. This process is verified by the significant reduction in the absorption peaks located at 1511 cm^−1^ versus 3520 cm^−1^ in the curves before and after curing PDMS+PU in Figure A1.

### 3.4. Mechanical Properties of Optimized Ceramics

**Figure 6:**
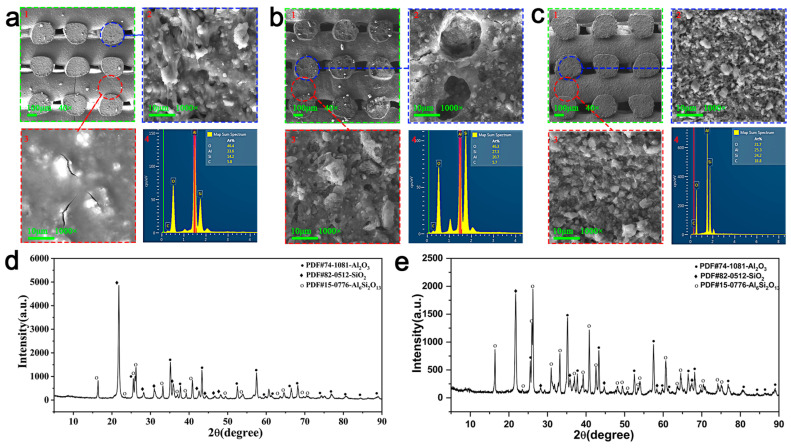
Reasons to use argon-atmosphere sintering for ceramic precursors and the main components of the ceramics obtained by sintering. (**a**) The microstructure of ceramic obtained by sintering with the PU-free Type I slurry in air. 1 shows the macroscopic structure at a magnification of 40 times, 2 shows the internal structure of the ceramic at a magnification of 1000 times, 3 shows the surface structure of the ceramic at a magnification of 1000 times, and 4 shows the percentage of each element in the ceramic EDS scanning results, the same as (**b**,**c**). (**b**) The microstructure of ceramic prepared from the PU-containing Type I slurry sintered in air. (**c**) The microstructure of ceramic prepared from the PU-containing Type I slurry sintered in an argon atmosphere. (**d**) XRD compound diffraction peaks of ceramics without polyurethane addition. (**e**) Diffraction peaks of ceramic XRD compounds with the addition of polyurethane.

Figure 6a,b show the electron microscope images of the ceramics obtained by sintering specimens without and with PU added in air, respectively. Comparing these images with the ceramic structure in Figure 6c, which was produced from the same material sintered in an argon atmosphere environment, reveals that the cross-section of the ceramic without PU Figure 6(a2) contained more pores, and its surface Figure 6(a3) had more crack defects or even fractures. According to the EDS elemental scanning results in Figure 6(a4,c4), pore generation manifested in a reduction in elemental C, which reacted with elemental O in the air. The cross-section in Figure 6(b2) of the PU-containing ceramic contains more pores, and its surface in Figure 6(b3) also has more crack defects. The introduction of PU and the gases generated during its thermal decomposition dilated the cracks and pores that already existed. These crack defects and pores worsened the mechanical properties of the material. Therefore, atmosphere sintering was used for the ceramic precursors in this study. Figure 6d,e show the results of the XRD analysis of the ceramic powder without and with PU addition, respectively. The addition of PU reduced the crystalline phase content of Al_2_O_3_ and SiO_2_, while the mullite crystalline phase was largely unaffected.

In order to test the feasibility of the optimization scheme, the three types of slurries were used to print 50 mm × 5 mm × 5 mm rectangular specimens, which were sintered in an argon atmosphere to form ceramics. Then, the mechanical properties of the ceramic specimens were tested using a universal testing machine. As ceramics are brittle materials and the ceramics prepared using this method are grid structures, the peak force of the ceramic specimens was tested by performing a three-point bending test. The bending strength of the specimens was obtained by the bending strength calculation formula, and the bending strength was compared between groups to determine the feasibility of the slurry optimization scheme.

The bending strength is calculated by the following formula:

$$\sigma =\frac{3PL}{{2bh}^{2}}$$

**Figure 7:**
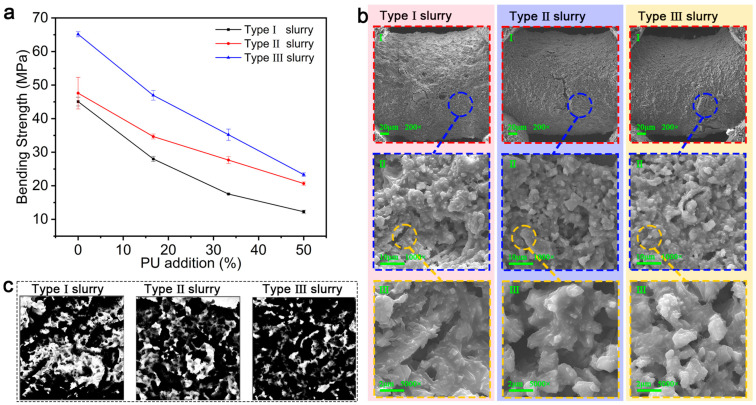
Mechanical property tests and microscopic morphologies of the three types of slurries. (**a**) Comparison of the bending strength of the three types of slurries with different PU contents. (**b**) Different magnifications of microscopic morphologies of the three types of slurries after sintering into ceramics at 1/2 PU addition, circles lead to localized zoomed-in results, I is the microstructure of each specimen under 200 times magnification, II is the microstructure of each specimen under 1000 times magnification, and III is the microstructure of each specimen under 5000 times magnification. (**c**) Quantitative plot of porosity for the three types of slurries at 1/2 the PU addition; the white parts are pores.

The value of span (*L*) is 24 mm; peak force (*P*), width of specimen (*b*), and height of specimen (*h*) are taken as in Table A1; and the calculation of the bending strength is shown in Figure 7a.

The statistical results of the bending strength for each type of slurry with different PU contents are shown in Figure 7a. The curing process for SE1700 was condensation curing, during which small molecule by-products (e.g., water and ethanol) were produced, which can cause microporosity in the ceramic specimen; after the introduction of PU, its thermal decomposition products (e.g., CO, CO_2_, NH_3_, HCN) increased these small porosity defects. As a result, the mechanical properties of the Type I slurry were relatively poor, and those of the ceramic specimens worsened with the increase in the PU content. As can be seen in Figure 7b, the ceramic specimens produced with the Type I slurry had the most pores on the surface and the loosest internal structure. With the introduction of DC184, which underwent addition curing, no by-products were produced; so, compared with the Type I slurry, the Type II slurry produced smaller defects, and the mechanical properties of ceramics without PU were better. With the introduction of PU, the expandable defects were relatively small, so the mechanical properties are better compared with those obtained with the same amount of PU introduced into the Type I slurry. Comparing the ceramic specimens prepared with the Type III and Type II slurries indicates that the ceramic prepared with the Type III slurry possessed a denser microstructure due to the increased solid-phase content of the ceramic powder. However, as seen in Figure 7a, with the increase in PU content, the mechanical properties deteriorated rapidly for this type of ceramic. The reason for this is that the crosslinking density of DC184 is higher than that of SE1700, the hardness of the cured DC184 is slightly higher than that of SE1700, and the increase in the solid-phase content of the Al_2_O_3_ nanoparticles reduced the tensile properties of the ceramic precursor. As a result, the hardness of the precursor specimen increased, its ability to adapt to deformation weakened, and with the increase in PU content, the gas generated by pyrolysis gradually increased, which made it easier to break the internal structure of the precursor before it was transformed into the ceramic product. A quantitative porosity map (as shown in Figure 7c) was obtained for the three types of slurries with a PU addition of 1/2 by analyzing the electron microscope images of the ceramic samples in Figure 7b, this enabled the quantification of the porosity levels of the three types of samples. The porosity of the above three ceramics was obtained by calculating the pore occupation ratio, in which the porosity of Type I slurry was 21.5% at 1/2 PU addition, Type II slurry was 17.3% at 1/2 PU addition, and Type III slurry was 15.5% at 1/2 PU addition. This observation aligns with the shifting trend depicted in Figure 7a and the preceding results. The introduction of DC184 led to an increase in ceramic density and a decrease in porosity, resulting in enhanced mechanical properties. Furthermore, an increase in the solid-phase content of alumina led to an additional enhancement in the ceramic density and mechanical properties. The collective outcomes of these experiments substantiate the viability of the proposed optimization scheme.

### 3.5. Deformability of Optimized Ceramics

**Figure 8:**
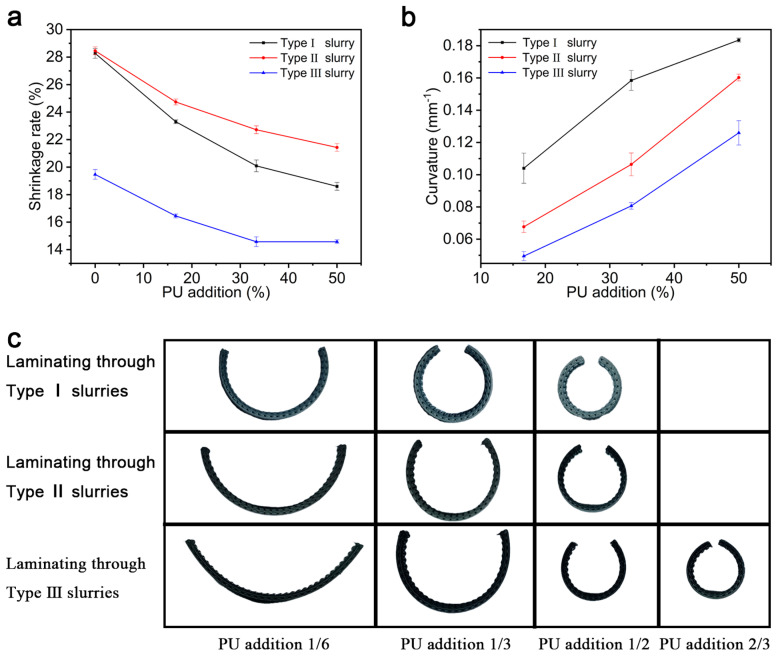
The deformability of the optimized precursors. (**a**) The shrinkage and contraction of the three types of slurries with different PU amounts incorporated. (**b**) The bendability of composite specimens with three types of slurries with different PU amounts incorporated. (**c**) The bending states of ceramics prepared via composite printing of the three types of slurries.

Figure 8a shows a graph of the variation patterns of the shrinkage and contraction rates of the ceramic specimens prepared with the three types of slurries with different PU contents. The ceramics prepared from the Type I and II base slurries were comparable in terms of shrinkage and contraction. As the amount of PU increased, the curvature of the change in the shrinkage rate of ceramics prepared from the Type II slurry was smaller than the others. The reason for this difference is that, after the introduction of DC184, the precursor produces fewer micropores during the curing process, the PU produces fewer defects during thermal decomposition, and the enhanced hardness of the precursor also hinders the extension of the defects, which confirms the change rule of the mechanical properties in Figure 7b. Increasing the solid-phase content of Al_2_O_3_ nanoparticles reduced the shrinkage space of the PDMS binder, so the shrinkage of ceramics prepared with the Type III slurry decreased significantly relative to those prepared with the Type II slurry. Figure 8b shows the curvature change rule of the ceramic specimens obtained by sintering with various types of base slurries and the addition of PU slurries using the basic deformation specimen preparation method in Section 3.2 for composite printing. It can be seen that the introduction of DC184 increased the hardness of the precursor while decreasing the deformability of the specimen, while the increase in the solid-phase content of Al_2_O_3_ nanoparticles again decreased the deformability. Figure 8c shows the physical bending of the specimens for a more intuitive visualization of the differences in the curvatures of the ceramic specimens. When increasing the solid-phase content of the Al_2_O_3_ nanoparticles, the PU content added to the Type III slurry for composite printing needed to be 2/3 to realize the same curvature produced by the Type I slurry when the added PU content was 1/2.

## 4. Conclusions

This study builds on the work on the polyurethane pyrolysis-driven 4D printing of ceramics, expanding and optimizing the scheme for improved performance. PU is introduced into the basic deformation scheme, and its thermal decomposition is utilized to achieve preliminary deformation in the pre-sintering stage. The pores created by the thermal decomposition of PU during the ceramization process reduce the shrinkage rate of the specimen, which drives the deformation of the ceramic precursor through an interlayer stress mismatch. The thermal treatment of the ceramics is accomplished in a single step without the introduction of additional inducing factors, and the preparation process is simple, efficient, and significantly improves the deformation capacity of the ceramic precursor. Moreover, no reverse springback occurs. This solves the problems of the low preparation efficiency and uneven stress distribution arising from the scheme of adopting mechanical means such as fixture fixation to realize the deformation of precursors, and there is no problem of reverse rebound arising from the adoption of hydrogel and shape memory materials. This scheme offers an excellent self-deformation capability and enables the preparation of ceramic specimens with large curvatures, through which ceramics with complex structures, such as scorpions and octopuses, were realized. A comparison of the sintering results of precursors in air and argon atmospheres revealed that sintering in an argon atmosphere results in ceramics with superior mechanical properties and more compact structures. By replacing part of PDMS SE 1700 in the original formulation with PDMS DC 184, the fluidity of the slurry was increased, which improved the incorporation capacity of the slurry for the ceramic powder and then increased the solid-phase content of the ceramic powder to improve the mechanical properties of the ceramic. The mechanical properties were also tested using a three-point bending test, and the feasibility of the scheme was verified by comparing the bending strength of the ceramic specimens prepared from the base slurry, the control slurry, and the modified slurry, and there is still room for improvement in terms of performance and accuracy when compared to conventional processes. The ceramic 4D printing preparation method in this scheme is efficient and convenient, and it is carried out in one step via high-temperature sintering without additional stimulation. The mechanical properties of the finished ceramic products are improved by the optimization of the slurry, which will extend the use of 4D-printed complex structural ceramics in engineering. Due to the introduction of PU, the ceramics prepared using this scheme have multistage pores and, as such, the deformed ceramics have potential applications in the field of thermal insulation materials.

## Data Availability

The original contributions presented in this study are included in the article. Further inquiries can be directed to the corresponding author.

## Abbreviations

The following abbreviations are used in this manuscript:

DIW	Direct Ink Writing
SLA	Stereo Lithography Apparatus
EPS	Expanded Polystyrene
PU	Polyurethane
PE	Polyethylene
PP	Polypropylene
PDMS	Polydimethylsiloxane
SE 1700	Dow Corning SE 1700 polydimethylsiloxane silicone rubber
DC 184	Dow Corning DC 184 polydimethylsiloxane silicone rubber
Al2O3	Alumina Nanoparticles
SEM	Scanning Electron Microscope
EDS	Energy Dispersive Spectrometry
XRD	X-Ray Diffraction
FTIR	Fourier Transform Infrared Spectrometer

## Appendix A

**Table A1:** Flexural experimental data from three sets of tests for each type of specimen.

Group I	Type I Slurry			Type II Slurry			Type III Slurry		
PU Addition	b (mm)	h (mm)	P(N)	b (mm)	h (mm)	P(N)	b (mm)	h (mm)	P(N)
0	3.58	3.15	45.19	3.59	3.16	42.04	4.04	3.55	92.92
1/6	3.84	3.38	34.56	3.76	3.31	40.64	4.18	3.68	75.99
1/3	4.01	3.53	24.17	3.87	3.41	35.25	4.26	3.75	55.61
1/2	4.08	3.59	18.46	3.92	3.45	27.27	4.28	3.76	39.23
**Group II**	**Type I Slurry**			**Type II Slurry**			**Type III Slurry**		
**PU Addition**	**b (mm)**	**h (mm)**	**P(N)**	**b (mm)**	**h (mm)**	**P(N)**	**b (mm)**	**h (mm)**	**P(N)**
0	3.52	3.12	41.46	3.58	3.15	50.36	4.01	3.53	89.20
1/6	3.81	3.36	32.38	3.75	3.29	38.69	4.16	3.65	69.94
1/3	3.98	3.50	23.90	3.85	3.39	32.54	4.25	3.73	60.37
1/2	4.04	3.56	16.71	3.91	3.43	25.65	4.25	3.73	37.42
**Group III**	**Type I Slurry**			**Type II Slurry**			**Type III Slurry**		
**PU Addition**	**b (mm)**	**h (mm)**	**P(N)**	**b (mm)**	**h (mm)**	**P(N)**	**b (mm)**	**h (mm)**	**P(N)**
0	3.57	3.13	44.58	3.57	3.16	49.08	4.03	3.53	91.33
1/6	3.82	3.37	34.37	3.76	3.32	39.37	4.16	3.67	73.26
1/3	4	3.51	24.06	3.86	3.4	35.06	4.26	3.74	58.71
1/2	4.07	3.56	17.79	3.91	3.43	26.68	4.25	3.75	39.57
